# Single-cell sequencing reveals the role of aggrephagy-related patterns in tumor microenvironment, prognosis and immunotherapy in endometrial cancer

**DOI:** 10.3389/fonc.2025.1560625

**Published:** 2025-03-25

**Authors:** Yuquan Yuan, Chunyan Ren, Jin Shu, Keyang Zhu, Ganghui Li, Bao Liu, Jianrong Huang, Yinde Huang, Chengzhi Zhao

**Affiliations:** ^1^ Department of Gynecologic Oncology, Chongqing Health Center for Women and Children, Chongqing, China; ^2^ Department of Gynecologic Oncology, Women and Children’s Hospital of Chongqing Medical University, Chongqing, China; ^3^ Chongqing Key Laboratory of Translational Research for Cancer Metastasis and Individualized Treatment, Chongqing University Cancer Hospital, Chongqing, China; ^4^ Clinical Medical College, North Sichuan Medical College, Nanchong, Sichuan, China; ^5^ Chongqing General Hospital, Chongqing University, Chongqing, China

**Keywords:** endometrial cancer, aggrephagy, tumor microenvironment, immunohistochemistry, prognosis

## Abstract

**Background:**

As a type of autophagy, aggrephagy degrades the aggregation of misfolded protein in cells and plays an important role in key genetic events for various cancers. However, aggrephagy functions within the tumor microenvironment (TME) in endometrial cancer (EC) remain to be elucidated.

**Methods:**

A total of 36,227 single cells from single-cell RNA-seq data derived from five EC tumor samples were comprehensively analyzed using a nonnegative matrix factorization (NMF) algorithm for 44 aggrephagy-related genes. Bulk RNA-seq cohorts from public repositories were utilized to assess the prognostic value of aggrephagy-related TME clusters and predict immune checkpoint blockade immunotherapeutic response in EC.

**Results:**

Fibroblasts, macrophages, CD8+T cells, and lymphatic endothelial cells were categorized into two to five aggrephagy-related subclusters, respectively. CellChat analysis showed that the aggrephagy-related subtypes of TME cells exhibited extensive interactions with tumor epithelial cells, particularly for macrophages. Moreover, aggrephagy regulators may be significantly associated with the pseudotime trajectories of major TME cell types as well as the clinical and biological features of EC. Bulk-seq analysis showed that these aggrephagy-related subclusters had significant predictive value for the survival and immune checkpoint blockade response in EC patients. Notably, immunohistochemical staining results manifested that the TUBA1A+ macrophage subtype was linked to less lymph node metastasis and longer survival, which were consistent with the bioinformatics analysis findings.

**Conclusions:**

This study provided a novel view of aggrephagy signaling in the EC tumor microenvironment, and intervention of aggrephagy was expected to improve the survival rate of EC patients.

## Introduction

Endometrial cancer (EC) is a malignancy that arises from the lining of the uterus (1). The incidence of EC in 2022 was 420,242 worldwide, and EC is the sixth most commonly occurring female cancer. EC has been broadly divided into two types by histology and clinical outcomes (2–4). Type I tumors comprise the large majority of grade I or grade II endometrioid adenocarcinomas, are associated with estrogen excess, hormone-receptor positivity, and obesity, and are often preceded by endometrial hyperplasia (5, 6). Type II tumors are more common in older, non-obese women, primarily including grade III endometrioid adenocarcinomas, undifferentiated, serous clear cells, and carcinosarcomas. Type II tumors are typically estrogen-independent, arising in atrophic endometrium from intraepithelial carcinoma, and are related to a poorer prognosis (7, 8). Surgery is the primary treatment for EC, but 15-20% of patients continue to relapse after surgery (9). Additionally, chemotherapy and radiotherapy tend to fail and are estimated to benefit only 10-15% of recurred patients (10). The median survival rate of patients with recurred or metastatic EC is less than 1 year (11). There remains a lack of effective treatment options for patients with advanced or terminal-stage EC. Therefore, identifying new potential molecular targets for diagnosing and treating EC is of critical clinical importance.

As tumors progress and metastasize, tumor cells typically demonstrate elevated metabolic activity and increased protein synthesis. The synthesis and degradation of proteins are closely related to the biological functions of tumor cells (12). When under various internal and external stress factors, the imbalance in protein degradation leads to the accumulation of misfolded proteins, forming protein aggregates (13). Abnormal functional protein aggregation can further affect downstream signal transduction and eventually promote tumor progression (14). In eukaryotic cells, the ubiquitin-proteasome system (UPS) is the primary pathway for eliminating misfolded proteins (15). SPOP is an adaptor protein of the CUL3-RBX1 E3 ubiquitin ligase complex, which mediates targeted ubiquitination and proteasomal degradation of specific proteins (16). Notably, SPOP mutations (5.7-10%) identified in EC impair UPS function, leading to toxic protein accumulation that drives tumor progression (17, 18). Aggrephagy, a form of selective autophagy, can degrade specific abnormal protein aggregates, inclusion bodies, and other structures, serving as a crucial pathway for cells to eliminate misfolded proteins (19). By efficiently removing abnormal protein aggregates, aggrephagy protects cells from their toxic damage and maintains the balance of protein degradation (20). Thus, as a therapeutic approach against protein aggregates, aggrephagy is gaining increasing attention and offers new perspectives for tumor treatment (14, 21–23).

The tumor microenvironment (TME) is a dynamic and complex system composed of tumor cells, immune cells, stromal cells, extracellular matrix, an extensive vascular network, and other secreted factors (24). The infiltrating immune cells include T cells, B cells, and macrophages; the stromal cells mainly include tumor-associated fibroblasts (CAFs) and endothelial cells (25, 26). Numerous studies have confirmed that the TME in which tumor cells reside plays a crucial role in the initiation, progression, and metastasis of tumors (27). Single-cell RNA sequencing (scRNA-Seq) is a novel technology that amplifies, sequences, and analyzes the RNA transcriptome of individual cells at the single-cell level, allowing exploration of each cell’s gene expression and functional state (28). This technology enables a comprehensive analysis of the complex interaction networks between tumor cells and the tumor microenvironment within the TME from a single-cell perspective (29). Moreover, scRNA-Seq can further investigate the intricate intercellular communication between different cell subtypes in the TME, providing a theoretical foundation for exploring tumor formation, progression, and treatment.

Previous research indicated that macrophages can increase the resistance of EC to radiotherapy, and CAFs promote the rapid progression and metastasis of EC by participating in intercellular communication (30, 31). The infiltration degree of various T cell subgroups plays a crucial role in predicting the prognosis of EC (32). However, little research has reported the cell-cell interaction between aggrephagy-related subtypes of TME cells and tumor cells. Understanding the interactions and molecular mechanisms between aggrephagy-related subtypes and the tumor cells can provide deeper insights into EC and guide diagnosis and treatment.

In this study, we used scRNA-seq to explore how aggrephagy affects key TME cells, including T cells, macrophages, CAFs, and endothelial cells, in the occurrence, development, and treatment of EC. Through a comprehensive analysis of the microenvironment associated with EC, this study revealed that aggrephagy may affect EC progression by regulating intercellular communication within the TME.

## Materials and methods

### Study design and data collection

scRNA-seq data from five EC patients were sourced from GSE173682 in the Gene Expression Omnibus (GEO) database (https://www.ncbi.nlm.nih.gov/geo). Following sample integration and batch correction, we established a gene expression and phenotype matrix comprising 36,227 cells. Additionally, we collected bulk mRNA sequencing data and clinical records for 705 patients from The Cancer Genome Atlas (TCGA) and GEO databases, including TCGA-UCEC, GSE63678, GSE17025, and GSE115810 (**Supplementary File 1**). All datasets analyzed in this study are accessible via previous publications or in the public domain, which are vetted by their ethics committee.

### Visualization of EC sample cell types and subtype

We processed the scRNA-seq matrix using the Seurat package (v4.4.0) within the R environment. After generating the Seurat objects, we selected the top 2,000 genes as the most variable features. These features provided the basis for normalizing scRNA-seq data at the single-cell level, a task accomplished through the FindVariableFeatures function in Seurat. We applied the ScaleData and RunPCA functions to determine the number of principal components. Further dimensionality reduction was achieved using the t-SNE and UMAP methods, which effectively summarized the top principal components. Cell clustering was performed using the FindNeighbors (dimension = 15) and FindClusters (resolution = 0.8) functions. We utilized the respective gene expression levels of well-known marker genes to annotate the major cell types within EC. The primary marker genes include myeloid cells (C1QA, LYZ, CD68, CD1C, CPA3); epithelial cells (EPACAM, CDH1, KRT7, KRT19); fibroblasts (LUM, PDGFRA, PDGFRB, ACTA2); endothelial cells (VWF, CCL21, PECAM1); T/B cells (CD8A, IL7R, NKG7, CD3E, KLRD1, CD79A); and smooth muscle cells (MYH11, MYLK).

### Pseudotime trajectory analysis of aggrephagy gene for TME cells

Monocle package (v2.22.0) was applied for scRNA-seq data to explore the correlation of aggrephagy regulators and pseudotime trajectories (33). The highly variable genes were defined using the following criteria: mean expression levels ≥ 0.1 and empirical dispersion ≥ 1 * dispersion fit. Subsequently, we used the ‘plot_pseudotime_heatmap’ function to generate heatmaps that display the dynamic expression of aggrephagy regulators in the pseudotime trajectories of different TME cell types in EC.

### Non-negative matrix factorization of aggrephagy−related genes in TME cells

To explore how aggrephagy affected the different cell types within the TME, a list of 44 aggrephagy-related genes was downloaded from the molecular signature database (**Supplementary File 2**). Subsequently, we conducted a dimension reduction analysis for aggrephagy-related genes in all TME cell types. To balance computational scalability and resolution, we first applied principal component analysis (PCA) to identify major cell clusters. Subsequent, the non-negative matrix factorization (NMF) was employed to identify different cell subtypes within these cell types, according to the scRNA expression matrix. These analytical procedures strictly adhered to established methodologies in previous studies (34).

### Identification of marker genes for aggrephagy−related cell subtypes within TME cells

The FindAllMarkers function was applied to list the markers of each NMF cluster within EC. To select characteristic genes, we set the log fold change (logFC) threshold to 0.5. If a gene with logFC greater than 1, prioritize aggrephagy-related genes that ranked highest in the list. We utilized the DotPlot function to show the top-ranking genes with the highest expression levels in each NMF cluster. The FeaturePlot function was used to illustrate the distribution of specific aggrephagy genes within the NMF clusters in the TME of EC.

### Cell−cell communication analysis

We used the CellChat package (v1.6.1) to generate CellChat objects and facilitate the analysis of intercellular communication. The ligand-receptor interactions were utilized to find communication patterns between different cell types. We projected the ligands and receptors into the protein-protein interaction network to identify cell communication events. Besides, we specifically explored communication between subtypes of aggrephagy-related cells and epithelial cells.

### NMF aggrephagy−related subtypes functional enrichment analysis

We used the clusterProfiler package (v4.2.2) to detect the Kyoto Encyclopedia of Genes and Genomes (KEGG) and the Reactome pathway databases based on the marker genes of the aggrephagy clusters in TME cell types. The CytoScape enrichment map function was applied to visualize these pathways. Gene sets with an adjusted p-value of < 0.05 were considered as significantly enriched. We used the scMetabolism package (v0.2.1) to evaluate the activity of cell metabolic pathways.

### NMF aggrephagy−related subtypes SCENIC analysis

We used The SCENIC package (v1.3.1) to explore the gene regulatory network involving transcription factors (TFs) in EC (35). Two gene motifs, hg19-tss-centered-10 kb and hg19–500 bp-upstream from the RcisTarget database, were applied to detect the transcription start site and explore gene regulatory networks within the scRNA-seq data of OS. TFs with adjusted p-values corrected by the Benjamini–Hochberg method of less than 0.05 were used for further analysis.

### Survival analyses with aggrephagy−related signatures in public bulk RNA−sequence datasets

Based on the FindAllmarker function, we generated aggrephagy-related gene signatures for all NMF cell clusters. We also calculated the main cell types within the EC TME based on the scRNA data. Then, we used the GSVA function to compute these gene signature scores in public EC datasets. To explore the relationship between the aggrephagy-related NMF signatures and the overall survival rate of EC patients, we conducted the log-rank test and Cox proportional hazard regression analysis. The cutoff values of different NMF cell signatures were calculated using the Survminer package (v0.4.9) to plot Kaplan–Meier curves.

### Immunotherapy analysis

To forecast the responses of EC patients to immune checkpoint blockade (ICB) immunotherapeutic, we uploaded expression data to the Tumor Immune Dysfunction and Exclusion (TIDE) website. After obtaining the output data, we analyzed the connections between aggrephagy-related NMF signatures. Additionally, immune checkpoints extracted from public datasets were assessed against the respective dataset.

### Immunohistochemistry semiquantitative analysis

Tissue sections on the microarray were subjected to antigen retrieval using Tris-EDTA (pH 9.0) for 20 minutes. After returning to room temperature, sections were treated with 3% hydrogen peroxide to block endogenous peroxidase activity. Subsequently, sections were incubated with either an anti-TUBA1A antibody (1:100; Servicebio, Wuhan, China) or an anti-CD86 antibody (1:100; Servicebio, Wuhan, China) overnight at 4°C. The next day, after reaching room temperature, secondary antibodies conjugated to either HRP or a fluorophore were applied and incubated for 1 hour at room temperature. After washing with PBS, tissue sections were developed using DAB for immunohistochemistry or stained with DAPI for immunofluorescence. The slides were then scanned using an Olympus microscope (Tokyo, Japan). Intensity Scoring: Cell staining intensity is scored on a four-tier scale: 0 points for no positive staining (negative), 1 point for light yellow (weakly positive), 2 points for brownish yellow (positive), 3 points for dark brown (strongly positive). Percentage of Positive Cells: The percentage of positive cells is also graded on a four-tier scale: 1 point for ≤25%, 2 points for 26%-50%, 3 points for 51%-75%, and 4 points for >75%. The final score is calculated by multiplying the intensity score by the percentage score (36). Immunofluorescence: Semiquantitative analysis for immunofluorescence is performed according to methodologies described in previous literature (37).

### Statistical analysis

The standard tests included the Student’s t-test, Wilcoxon rank-sum test, Kruskal–Wallis test, and Chi-square test for continuous target or category variables. The log-rank test was used for survival analyses. Statistical analyses of this study were conducted in R 4.1.3 software, and a two-sided p-value < 0.05 was regarded as statistically significant.

## Results

### Single cell landscape of EC

**Figure 1:**
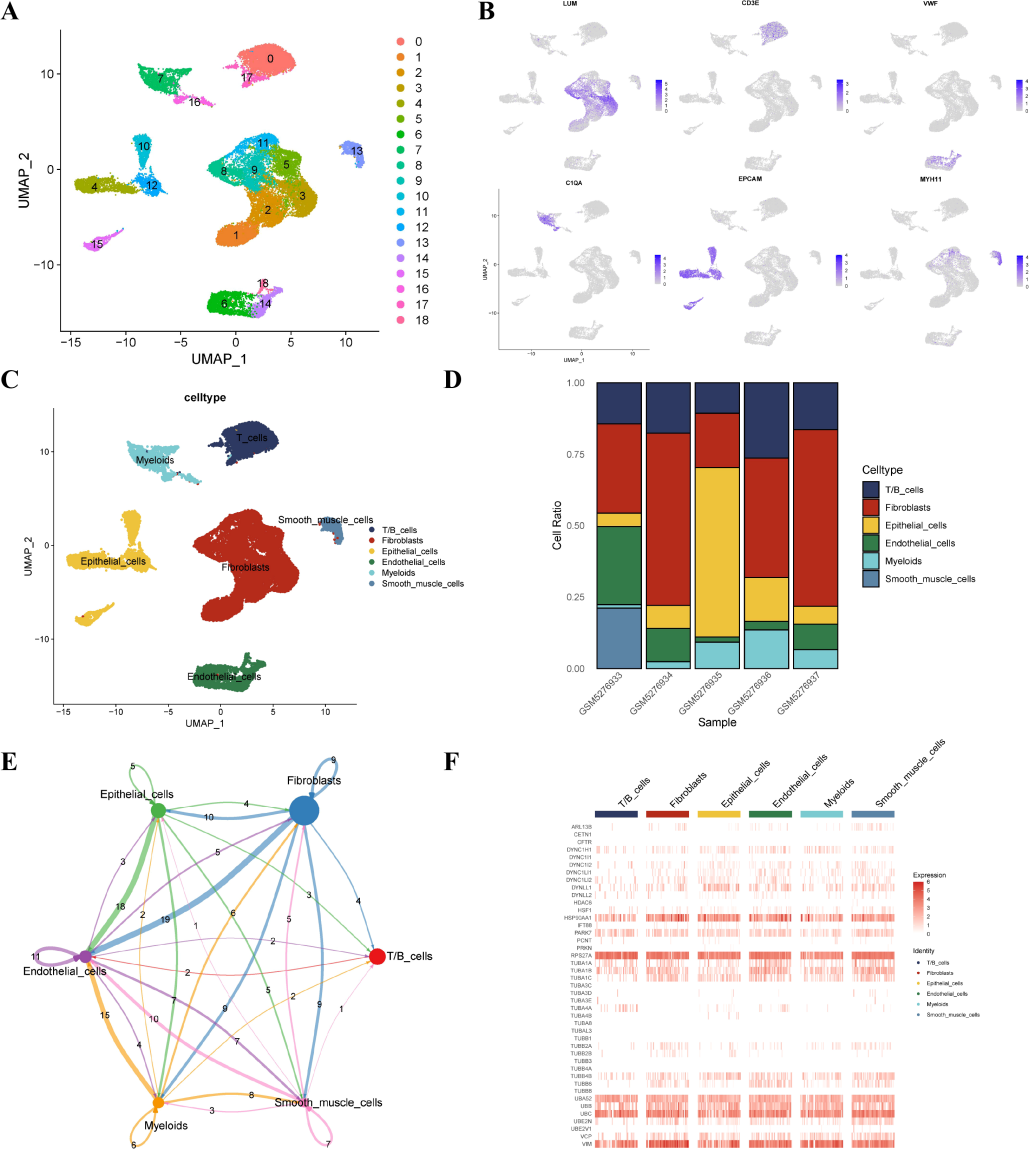
Overview of aggrephagy-related genes in the single-cell data for EC. **(A)** Dimensionality reduction clustering of EC samples. **(B)** Annotated marker genes for major cell types. **(C)** UMAP plot highlighting the six main cell clusters in EC. **(D)** Cell composition within each EC sample. **(E)** Cell–Cell communications between the main six cell types by Cell chat analysis. **(F)** Heatmap illustrating aggrephagy-related gene distribution in major cell types.

Dimensionality reduction and annotation of single cells for five EC samples were conducted to explore the TME and cellular diversity. We set the gene expression range for each single cell between 200 and 4,000, resulting in 29,667 cells. UMAP analysis was performed based on the differentially expressed genes of all cells, and 19 clusters were found (**Figure 1A**). One marker gene of each cluster was selected for visualization (**Figure 1B**). We annotated each cluster and identified six main clusters, including myeloid cells, epithelial cells, CAFs, endothelial cells, T/B cells, and smooth muscle cells according to previously reported cell marker genes (**Figure 1C, D**). **Figure 1D** visualizes the proportion of different cell types in each sample. Cell-chat analysis showed complex cell communications among these cell types (**Figure 1E**). The epithelial cells have apparent communication with endothelial cells, T/B cells, CAFs, and myeloid cells. Subsequently, we generated the heat map to show the expression of the aggrephagy-related genes in different cell types (**Figure 1F**). Several aggrephagy-related genes were highly expressed in the six cell types, including RPS27A, HSP90AA1, UBC, TUBA1A, and VIM (**Figure 1F**).

### Novel aggrephagy−related CAFs contributed to the TME of EC

**Figure 2:**
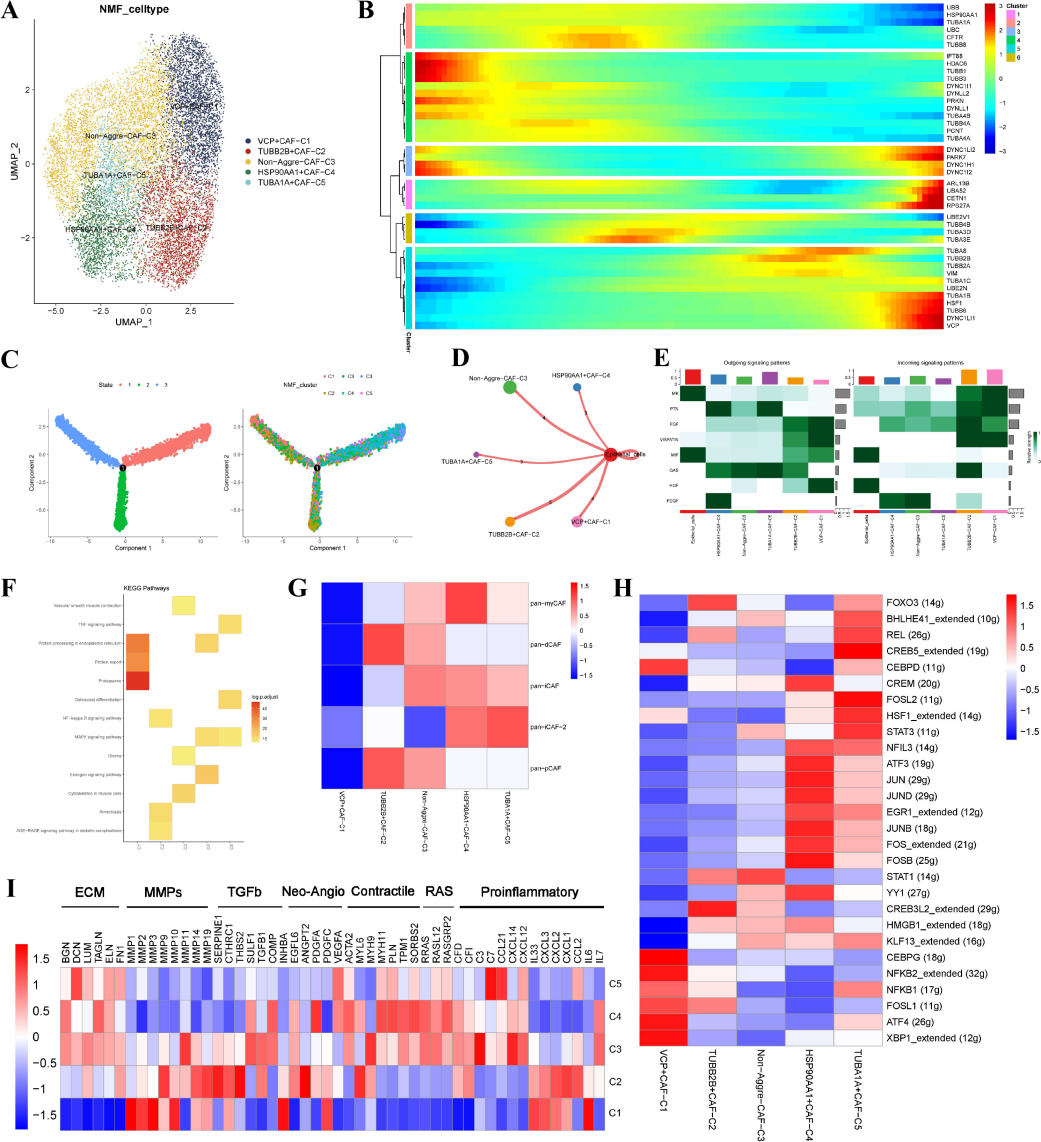
Identification of novel aggrephagy-related CAFs in the TME of EC. **(A)** UMAP plot showing five subtypes of aggrephagy-related NMF CAFs. **(B)** Pseudotime trajectory analysis illustrating aggrephagy-related gene dynamics in CAFs. **(C)** The developing status of CAFs NMF clusters obtained in pseudotime analysis. **(D)** Cell–Cell communications from aggrephagy-related CAFs to epithelial cells. **(E)** Relative strength of enriched outgoing and incoming signals in aggrephagy-related CAFs and epithelial cells. **(F)** Heatmap showing activated KEGG pathways in aggrephagy-related CAFs by using the DEGs among these groups (p < 0.05). **(G)** Association between CAF subtypes and aggrephagy-related CAFs. **(H)** Heatmap displaying differential TFs activities across five aggrephagy-related CAFs. **(I)** Heatmap showing the different average expression of common signaling pathway genes in the five aggrephagy-related CAFs.

We conducted dimensionality reduction and NMF clustering for 13,004 CAFs and found five new CAF subtypes (**Figure 2A**). The pseudotime analysis showed that the aggrephagy-related genes had an important role in the trajectory process of CAFs (**Figures 2B, C**). The cell-cell communication analysis showed that aggrephagy-related fibroblast clusters and epithelial cells had different numbers of ligand-receptor links (**Figure 2D**). The activation state of the signal pathway showed that VCP+CAF-C1 and TUBB2B+CAF-C2 subclusters interacted more with epithelial cells (**Figure 2D**). Among the signaling molecules, the epithelial cells mainly sent MIF and MK, while the VCP+CAF-C1 and TUBB2B+CAF-C2 subcluster mostly received MK, PTN, FGF and VISFATIN (**Figure 2E**). The KEGG enrichment analysis revealed that the VCP+CAF-C1 cluster exhibited obvious protein export, proteasome, and protein processing in the endoplasmic reticulum pathway (**Figure 2F**). We calculated the Pan-CAF signatures reported in a previous study and detected that the VCP+CAF-C1 cluster was negatively associated with any CAF subtype (38). By contrast, the TUBB2B+CAF-C2, HSP90AA1+CAF-C4, and TUBA1A+CAF-C5 were strongly associated with desmoplastic CAF (pan-dCAF), myofibroblast-like CAF (pan-myCAF), and inflammatory CAF (pan-iCAF), respectively (**Figure 2G**). According to the gene regulatory network analysis, significant differences were observed among five clusters of CAF subtypes. In the VCP+CAF-C1, HSP90AA1+CAF-C4, and TUBA1A+CAF-C5 clusters, several TFs, such as CREB5, FOSL2, STAT3, JUND, NFKB1, and CEBPG, were significantly upregulated. However, in the TUBB2B+CAF-C2 and non-aggre-CAF-C3 clusters, most TFs did not show notable upregulation (**Figure 2H**). The expressions of the VCP+CAF-C1 and Non-aggre-CAF-C3 pathway genes were significantly different, as shown in the pathway heatmap (**Figure 2I**). These results indicated the heterogeneity of CAFs in EC and the significance of aggrephagy for the classification of CAFs.

### Identification of novel aggrephagy-related macrophages in the TME of EC

**Figure 3:**
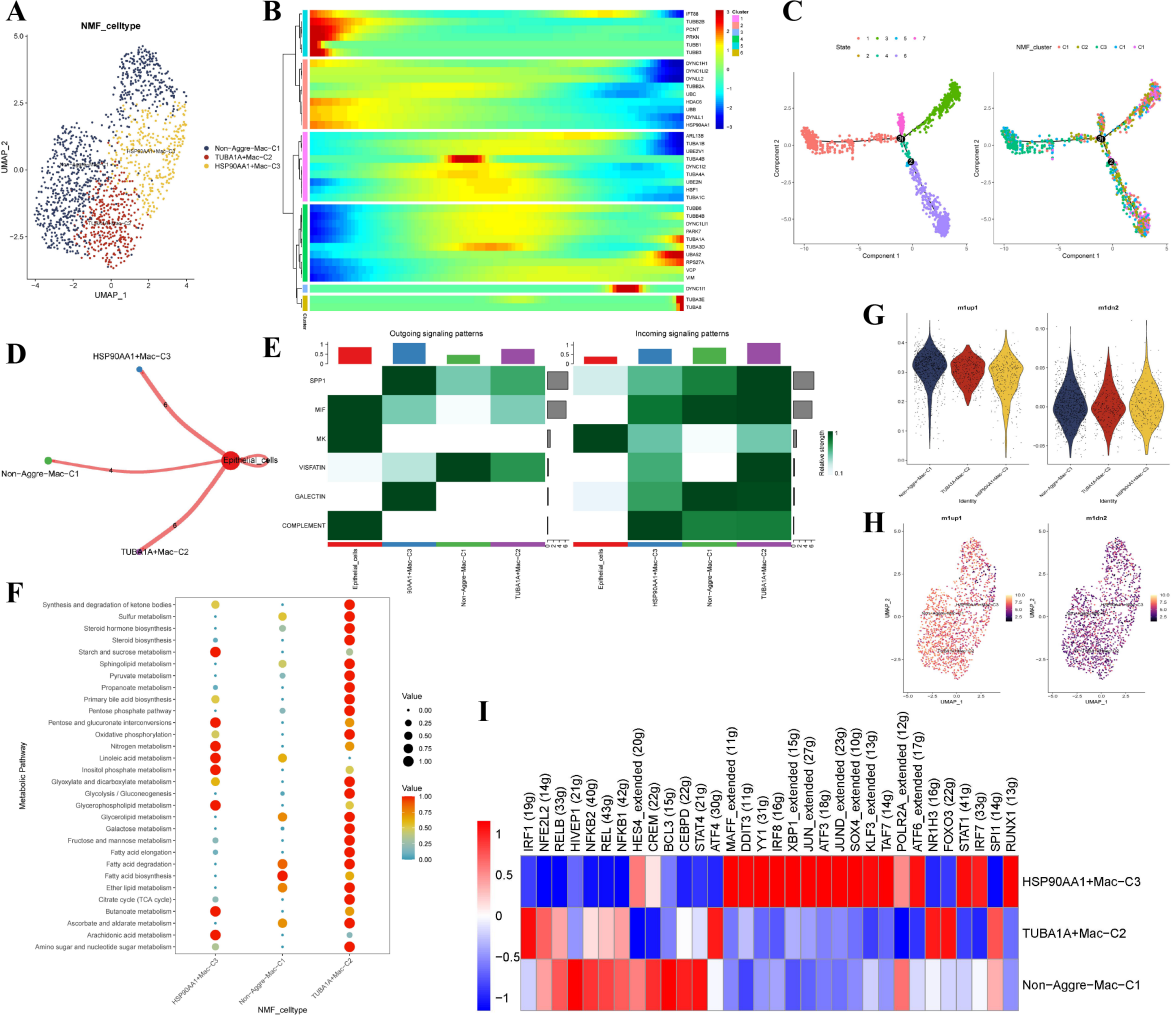
Identification of novel aggrephagy-related macrophages in the TME of EC. **(A)** UMAP plot showing three subtypes of aggrephagy-related NMF macrophages. **(B)** Pseudotime trajectory analysis illustrating aggrephagy-related gene dynamics in macrophages. **(C)** The developing status of macrophages NMF clusters obtained in pseudotime analysis. **(D)** Cell–Cell communications from aggrephagy-related macrophages to epithelial cells. **(E)** Relative strength of enriched outgoing and incoming signals in aggrephagy-related macrophages and epithelial cells. **(F)** Metabolic pathway analysis of aggrephagy-related macrophages subtypes. **(G)** M1 and M2-like phenotype scoring among different aggrephagy-related macrophage subtypes. **(H)** UMAP plot illustrating M1 and M2 activity across aggrephagy-related NMF macrophage subtypes. **(I)** Heatmap showing the different average expression of common signaling pathway genes in the three aggrephagy-related macrophage subtypes.

We extracted 1,473 macrophages from the myeloid cells (1,954 cells) (**Supplementary Figures S1A-D**). After further integrating the five macrophage NMF clusters, we get three main aggrephagy-related macrophage clusters named Non-Aggre-Mac-C1, TUBA1A+Mac-C2, and HSP90AA1+Mac-C3 (**Figure 3A**). The aggrephagy-related genes played a crucial role in the trajectory process of macrophages according to Pseudotime analysis (**Figures 3B, C**). Different number of ligand-receptor links between these aggrephagy-related macrophage clusters to epithelial cells were discovered by cell-chat analysis (**Figure 3D**). Additionally, the activation state of the signal pathway showed an obvious difference, and the TUBA1A+Mac-C2 subcluster interacted more with epithelial cells (**Figure 3E**). We conducted KEGG enrichment analysis to evaluate the relationship between aggrephagy-related macrophage clusters and special pathways and found 30 metabolic pathways enrichment results (**Figure 3F**). The metabolic pathways enrichment results showed that TUBA1A+Mac-C2 were activated in SPP1, MIF, VISFATIN, and GALECTIN pathways (**Figure 3F**). No significant difference was observed between M1 and M2 macrophages among aggrephagy-related subclusters of macrophages (**Figures 3G, H**). SCENIC analysis showed different activations of TFs among macrophage subclusters. From the pathway heatmap, TFs of TUBA1A+Mac-C2 were mainly down-regulated, and HSP90AA1+ Mac-C3, Non-Aggre-Mac-C1 were opposite (**Figure 3I**). Overall, we identified novel aggrephagy-related macrophage subclusters and observed that these subclusters were comparatively more active in metabolism and transcription than non-aggrephagy-related subclusters.

### Contribution of aggrephagy−related CD8+T cell to TME of EC

**Figure 4:**
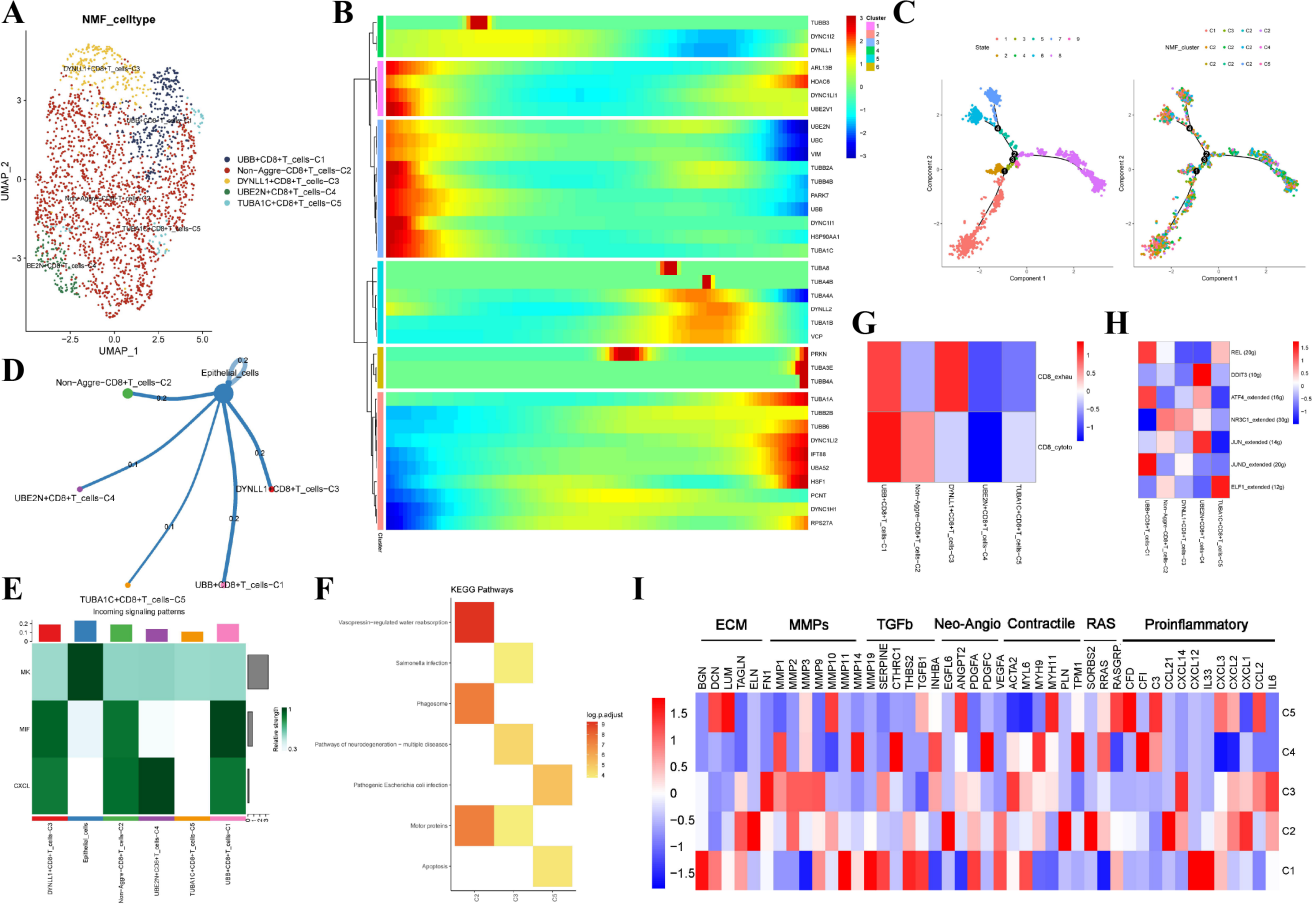
NMF clusters of aggrephagy-related genes in CD8+T Cells. **(A)** UMAP plot showing five subtypes of aggrephagy-related NMF CD8+T cells. **(B)** Pseudotime trajectory analysis illustrating aggrephagy-related gene dynamics in CD8+T cells. **(C)** The developing status of CD8+T cell NMF clusters obtained in pseudotime analysis. **(D)** Cell–Cell communications from aggrephagy-related CD8+T cells to epithelial cells. **(E)** Relative strength of enriched incoming signals in aggrephagy-related CD8+T cells and epithelial cells. **(F)** Heatmap illustrating activated KEGG pathways in aggrephagy-related CD8+T cells. **(G)** Heatmap showing the CD8+T cell function signatures (exhaustion score and T cytotoxic score) among five aggrephagy-related CD8+T cells subtypes. **(H)** Heatmap showing differential activities of TFs among five aggrephagy-related CD8+T cell clusters. **(I)** Heatmap showing the different average expression of common signaling pathway genes in the five aggrephagy-related CD8+T cell.

After further analysis, we identified six main cell types, including CD8+T cells, regulatory T cells, conventional T cells, NK cells, B cells, and plasma cells from the 5,077 T/B cells (**Supplementary Figures S2A-D**). Subsequently, 2,173 CD8+T cells were divided into 12 clusters by the NMF algorithm, and a total of five aggrephagy-related cell clusters were finally generated. The pseudotime analysis showed that aggrephagy-related genes play a role in the early and late stages of cell differentiation (**Figures 4B, C**). There were different numbers of ligand-receptor links between these aggrephagy-related CD8+T cell clusters and epithelial cells according to the results of cell-cell communication analysis (**Figures 4D, E**). Enrichment analysis with KEGG showed that DYNLL1+CD8+T cells-C1, non-Aggre-CD8+T cells-C2, and TUBA1C+CD8+T cells-C5 cluster exhibited different pathway activation, while the UBB+CD8+T cells-C1 and UBE2N+CD8+T cells-C4 did not show significant pathway activation (**Figure 4F**). Furthermore, aggrephagy-associated CD8+T cells exhibit inactive characteristics and variable T cell activity. Notably, UBB+CD8+T cells-C1 showed characteristics of both an active CD8_exhau subtype and a more pronounced CD8_cytoto subtype (**Figure 4G**). SCENIC analysis showed different activation of TFs among CD8+T cell subclusters. From the pathway heatmap, TFs of UBB+CD8+T cells-C1 and UBE2N+CD8+T cells-C4 were mainly up-regulated, and other subclusters were opposite (**Figure 4H**). The expressions of pathway genes of the five subclusters were also different, as shown in the pathway heatmap (**Figure 4I**). To summarize, we identified that specific aggrephagy-related CD8+T cells, such as UBB+CD8+T cells, showed increased activity in cellular interactions, TFs, and T-cell cytotoxicity.

### Identification of novel aggrephagy-related lymphatic endothelial cell in the TME of EC

**Figure 5:**
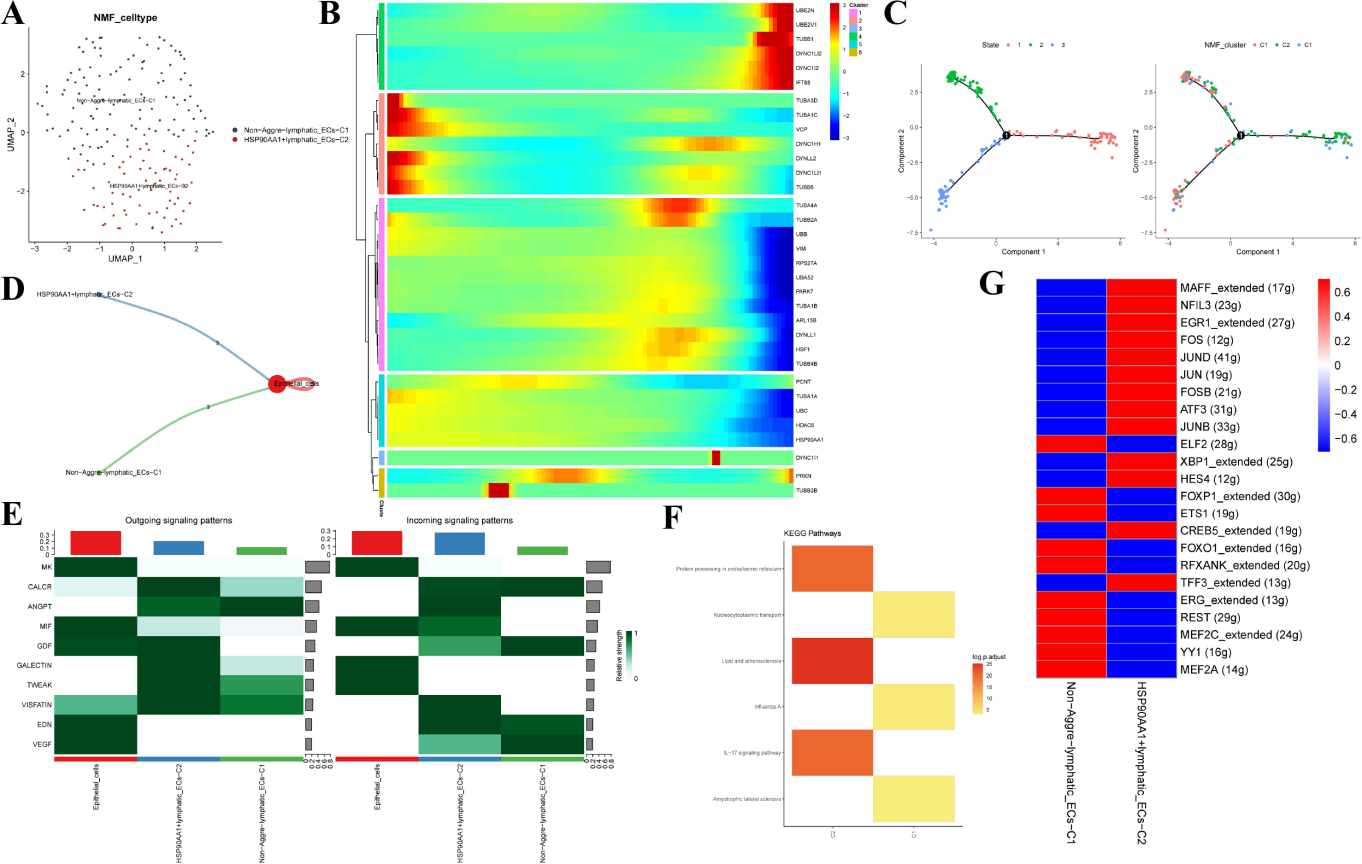
NMF clusters of aggrephagy-related genes in LECs. **(A)** UMAP plot showing two subtypes of aggrephagy-related NMF LECs. **(B)** Pseudotime trajectory analysis illustrating aggrephagy-related gene dynamics in LECs. **(C)** The developing status of LECs NMF clusters obtained in pseudotime analysis. **(D)** Cell–Cell communications from aggrephagy-related LECs to epithelial cells. **(E)** Relative strength of enriched outgoing and incoming signals in aggrephagy-related LECs and epithelial cells. **(F)** Heatmap showing activated KEGG pathways in aggrephagy-related LECs. **(G)** Heatmap displaying differential TFs activities across two aggrephagy-related LECs.

We found three main cell types including vein cells, artery cells, and lymphatic endothelial cells (LECs), from the 2,751 endothelial cells after further analysis (**Supplementary Figures S3A-D**). Lymph node metastasis is a common characteristic of EC. Therefore, LECs were divided into two aggrephagy-related cell clusters by the NMF algorithm (**Figure 5A**). The aggrephagy-related genes had an essential role in the trajectory process of LECs, varying in the early and late stages of cell differentiation (**Figures 5B, C**). Cell-cell communication analysis showed different numbers of ligand-receptor links between two aggrephagy-related LEC clusters and epithelial cells (**Figures 5D, E**). The KEGG enrichment analysis was conducted to explore the relationship between aggrephagy-related LEC clusters and special pathways (**Figure 5F**). HSP90AA1+lymphatic-ECs-C2 showed significant activation in protein processing in the endoplasmic reticulum and IL-17 signaling pathway (**Figure 5F**). SCENIC analysis showed obvious different activation of TFs between non-Aggre-lymphatic-ECs-C1 and HSP90AA1+lymphatic-ECs-C2 (**Figure 5G**).

### Aggrephagy−related TME patterns guide EC prognosis and immunotherapy

**Figure 6:**
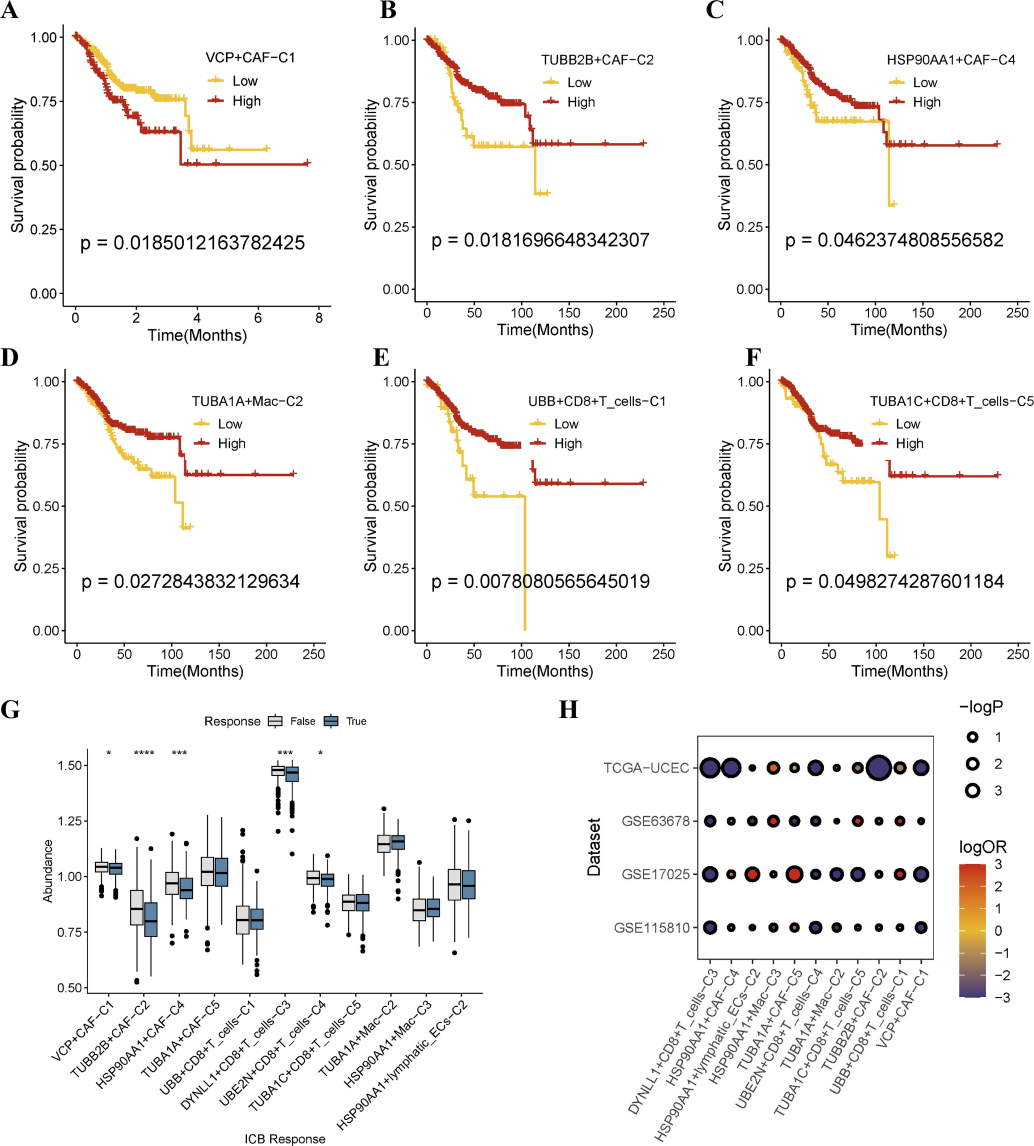
Overall of the prognosis and immunotherapy response of aggrephagy-related cell types in the bulk sequence from public cohorts. **(A–F)** Kaplan–Meier plot of different aggrephagy-related TME cell subtypes in TCGA-UCEC database. **(G)** Prediction of ICB immunotherapeutic response in TCGA-UCEC database. **(H)** ICB analysis among four different datasets. Significance levels are indicated as follows: * (P ≤ 0.05), *** (*P* ≤ 0.001), and **** (P ≤ 0.0001).

We recalculated the aggrephagy-related gene expression in bulk RNA and scRNA data to find the signature of the main EC TME cell types. Based on the DEGs of aggrephagy-related TME cells, we used the GSVA to calculate the aggrephagy sub-score in 548 EC patients downloaded from TCGA-UCEC and to explore their prognosis. All scores were divided into two groups for the cox regression analysis. Notably, as the variation in the aggrephagy genes across special aggrephagy sub-cell types, the overall survival rates of EC patients were significantly different among these sub-clusters, including CAFs, macrophages, and CD8+T cells (**Figures 6A-F**). Especially, we observed a positive correlation between the expression of TUBA1A+MAC-C2 and survival probability, which was similar to the results from the TCGA database (http://gepia2.cancer-pku.cn/) and the Kaplan-Meier Plotter online platform (https://kmplot.com/) (**Supplementary Figures S4A-B**). Subsequently, we used the transcriptomic biomarkers of EC patients to predict ICB immunotherapy response by the TIDE website (**Figures 6G, H**). The results of logistic regression analysis indicated that different TME cell subclusters contributed to the ICB response in EC patients in both the TCGA-UCEC, GSE17025, GSE115810, and GSE63678 databases (**Figures 6G, H**). According to our predictions, high expression of DYNLL1+CD8+T cells and UBE2N+CD8+T cells was associated with a worse ICB response.

### TUBA1A+Mac subtype is linked to less lymph node metastasis and longer survival

**Figure 7:**
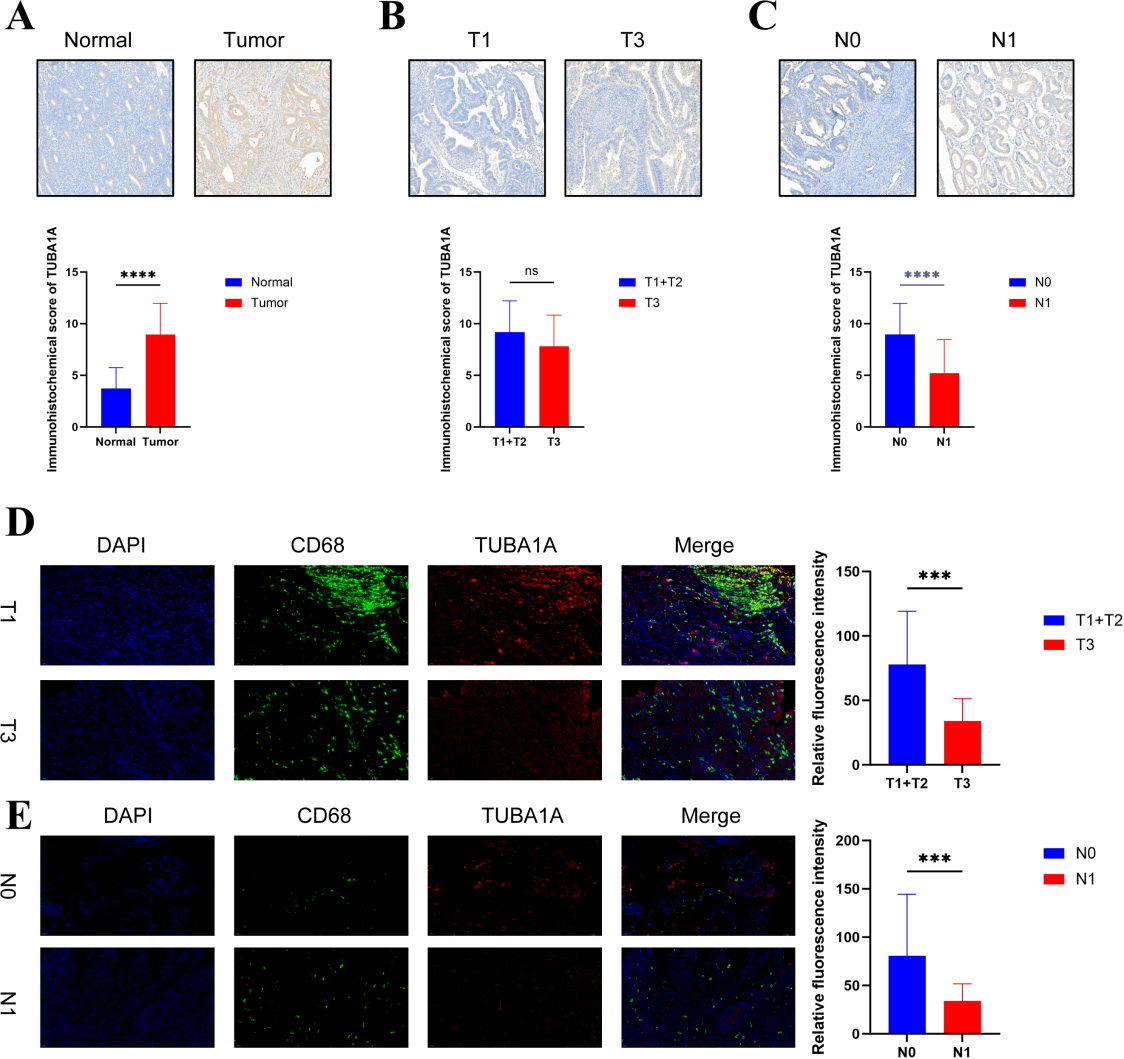
Validation of TUBA1A expression in EC. **(A–C)** The expression levels of TUBA1A by immunohistochemistry in normal vs. tumor tissues, T1 vs. T3 stages, and N0 vs. N1 lymph node status. **(D, E)** Immunofluorescence analysis showing the fluorescence intensity of TUBA1A and CD68 co-expressing cells in T1 vs. T3 stages and N0 vs. N1 lymph node status. Significance levels are indicated as follows: ns (not significant, P > 0.05), *** (*P* ≤ 0.001), and **** (P ≤ 0.0001).

In our analysis, TUBA1A was identified as a marker gene for specific subtypes of macrophages. TUBA1A+macrophages were associated with a better prognosis. Immunohistochemistry further revealed that TUBA1A is highly expressed in EC (p < 0.0001), with significantly higher expression levels observed in N0 stages (no lymph node metastasis, p < 0.0001). However, no statistically significant difference was observed between T1+T2 and T3 stages (p = ns) (**Figures 7A-C**). These findings suggested that TUBA1A expression increases during tumorigenesis but is associated with fewer lymph node metastases. Furthermore, immunofluorescence staining showed a significant increase in the relative intensity of CD68 and TUBA1A co-expressing cells in both T1+T2 stages (p < 0.001) and N0 status (p < 0.001) (**Figures 7D, E**). These results suggest that TUBA1A+Mac-C2 are associated with lower T-stage and are accompanied by fewer lymph node metastases. Overall, TUBA1A may exert a pro-tumorigenic effect, but its presence is linked to improved prognostic outcomes.

## Discussion

Autophagy is a tightly regulated and highly conserved lysosomal degradation pathway. It primarily consists of three main pathways, including chaperone-mediated autophagy, microautophagy, and macroautophagy (39–41). It is widely acceptable that autophagy deficiency or deregulation is associated with different human diseases, such as cancer, autoimmune, and neurodegenerative disorders (41). Several studies have explored the crucial role of autophagy in the pathophysiology of EC (42–45). However, no study has investigated the potential role of aggrephagy in EC, and even few reports of aggrephagy associated single cell level (46, 47). As a type of macroautophagy, aggrephagy can degrade specific abnormal protein aggregates, serving as a crucial pathway for cells to eliminate misfolded proteins (19). A deeper understanding of the specific mechanisms of action of aggrephagy in EC can provide insights for the advancement of drug research and treatments. In this study, we have comprehensively investigated aggrephagy-related genes of main cell types in the TME of EC and further identified the diversity of cell-cell interaction between aggrephagy associated TME cell subtypes and tumor cells, especially tumor epithelial cells at the 10X Genomic single-cell sequence level. We are able to understand how aggrephagy of these diverse cellular components of TME affects the clinical outcomes of individual EC patients by this unique and new perspective.

Cancer epithelial cells constitute the majority of tumor tissue and play a crucial role in the progression and biological behavior of EC (48). Within the complex and dynamic TME, cancer epithelial cells actively interact with various components, including immune cells, stromal cells, and endothelial cells (49). This interaction is bidirectional, with cancer epithelial cells both influencing and being influenced by their microenvironment. Understanding the intricate relationships within the TME is essential for developing more effective therapeutic strategies targeting. In this study, we discovered that the TME cells, including CAFs, macrophages, T cells and LECs all manifested the different aggrephagy regulatory patterns and the extensive communication with tumor epithelial cells. Furthermore, CellChat analysis showed that ligand-receptor pairs, the activation of angiogenesis related pathways further mediated the communication between aggrephagy-related subtypes of TME cells and tumor epithelial cells.

As one of the most important components of stromal cells, CAFs consist of pan-myCAFs, pan-iCAFs, pan-nCAFs, pan-dCAFs, and pan-pCAFs, according to specific molecular characteristics (38). In this study, we found that CAFs had complex cellular communication with cancer epithelial cells, especially the MIF-related pathway. Additionally, VCP+CAF-C1 and TUBB2B+CAF-C2 manifested more extensive communications with tumor epithelial cells compared with non-aggrephagy-associated CAFs. The pathway analysis results showed the participation of aggrephagy-related CAFs in critical signaling pathways, including the TNF signaling pathway, NF-kappa B signaling pathway, protein processing in the endoplasmic reticulum, proteasome, and protein export. Furthermore, the elevated expression of MMP factors was obviously increased in VCP+CAF-C1, such as MMP1, MMP2, MMP3, MMP9, and MMP10. Prognosis analysis has shown an adverse correlation between the expression and survival rate. Therefore, we speculated that VCP+CAF-C1 may disrupt the matrix barrier to promote the progress and metastasis of the tumor. Several studies have also revealed that elevated VCP expression is related to tumor progression and metastasis, including colorectal and breast cancers (50). The specific mechanisms of the VCP+CAFs subcluster in EC still require further research to be clearly elucidated.

Many studies have highlighted the crucial role of regulating and reprogramming immune cells in tumors (51, 52). We utilized the NMF algorithm to classify macrophages based on aggrephagy-related genes and discovered a significant phenomenon: aggrephagy-associated macrophage subtypes actively engaging in extensive cross-talk with tumor cells. TUBA1A+MAC-C2 and HSP90AA1+MAC-C3 had more extensive communications with tumor epithelial cells compared with non-aggrephagy-associated macrophages. Furthermore, the CellChat analysis revealed that the TUBA1A+MAC-C2 displays a significant activation of SPP1, MIF, VISFATIN, and GALECTIN pathways. The metabolic processes significantly impacted macrophage, thus influencing cancer progression and immune responses, including glucose metabolism, fatty acid metabolism, and glutamine utilization. We observed that the metabolism-related pathways of aggrephagy-associated macrophage, especially in the TUBA1A+MAC-C2, were notably activated, such as fatty acid degradation, Citrate cycle (TCA cycle), and Propanoate metabolism et al. Notably, prognosis analysis has discovered a positive correlation between the expression of TUBA1A+MAC-C2 and survival probability. According to the results from the TCGA database and the Kaplan-Meier Plotter online platform (https://kmplot.com/), high expression of TUBA1A is also associated with improved survival rates in EC. Subsequently, we performed immunohistochemical staining for TUBA1A and macrophages to further investigate the role of TUBA1A+MAC-C2 in EC. Interestingly, the immunohistochemical staining results were consistent with the findings of our bioinformatics analysis, showing that TUBA1A+MAC-C2 is highly expressed in tumor tissues. Additionally, EC patients with high expression of TUBA1A+MAC-C2 had fewer lymph node metastases and better survival outcomes. Studies have shown that TUBA1A promotes macrophage infiltration in gastric cancer (53). The pro- and anti-tumor activities of macrophages in tumors are often closely linked to their polarization states. We hypothesize that TUBA1A may play a role in mediating the effects of macrophage polarization on lymph node metastasis in EC. Previous studies also found that overexpression of TUBA1A can reduce the migration and invasion abilities of cervical cancer cells (54). In the future, we will conduct additional experiments to explore how TUBA1A+MAC-C2 influences lymph node metastasis and prognosis in EC.

In addition to macrophages, we also identified that aggrephagy-associated CD8+T cells are involved in complex interactions with tumor cells. Furthermore, aggrephagy-associated CD8+T cells exhibit inactive characteristics and variable T cell activity. UBB+CD8+T cells robustly activated the cell cycle pathway, upregulated immune-related genes, and exhibited characteristics of both an active CD8_exhau subtype and a more pronounced CD8_cytoto subtype. Survival analysis revealed that UBB+CD8+T cells demonstrated a better survival rate in TCGA data. This is consistent with previous studies, which showed that CD8 cytotoxic T cells are associated with a positive impact on anti-tumor immune responses. These findings highlight the crucial role of aggrephagy in immune evasion and the tumor-restraining effect of macrophages and T cells.

Lymph node metastasis is the most common form of metastasis in EC, and LECs play a key role in this process (7, 55). The crosstalk between LECs and tumor cells appears to promote lymphangiogenesis, providing a pathway for tumor cells to invade the lymphatic system and disseminate to distant sites. Moreover, LECs contribute to creating a pre-metastatic niche by modulating the tumor microenvironment and facilitating immune evasion, thus enhancing the potential for tumor progression and metastasis (56). Understanding the role of LECs in EC may open up new avenues for targeted therapies to prevent or mitigate lymphatic spread. In this study, we discovered that LECs had complex cellular communication with cancer epithelial cells and HSP90AA1+lymphatic-ECs-C2 showed significant activation of MIF, GDF, VISFATIN, TWEAK, and GALECTIN pathways. Pathway analysis showed the participation of aggrephagy-associated LECs in essential signaling pathways, including Protein processing in the endoplasmic reticulum, Lipid and atherosclerosis, and IL-17 signaling pathway. However, survival analysis revealed that the expression of HSP90AA1+lymphatic-ECs-C2 had no significant impact on the prognosis of EC.

TFs play a crucial role in forming transcription initiation complexes, influencing transcription processes, and subsequently regulating downstream gene expression. Therefore, we analyzed TFs at the single-cell level to identify cell-specific gene regulatory networks. Each subtype of CAFs, macrophages, LECs, and CD8+T cells showed different TF characteristics. The VCP+CAFs exhibited a unique TF gene signature, such as NFKB2, NFKB1, FOSL1, CEBPD, ATF4, XBP1, and CEBPG. Notably, previous studies have discovered that RUNX1 and NR2F2 play an important role in tumor growth and metastasis, and the VCP+CAFs also demonstrated a worse survival rate (57–59). Furthermore, we also discovered the higher activity of FOXO3 and NR1H3 on TUBA1A+Mac-C2. Similarly, the correlation between tumor inhabitation and FOXO3 and NR1H3 was reported in previous research (60–62). Moreover, for LECs and CD8+T cells, we also found distinct TF characteristics of aggrephagy-associated cell subtypes. Based on these findings, we supposed that aggrephagy cell subtypes might influence specific TF regulatory networks to reshape and reprogram TME. Moreover, cell network analysis indicated that these aggrephagy-related TME cells were closely connected and interacted with tumor cells. The aggrephagy CAFs, LECs, and immune cell subtypes exhibited increased communication with cancer epithelial cells, suggesting that the regulation of the TME, and possibly the development of immune suppression, may be partially influenced by aggrephagy.

Given the complex internal patterns of aggrephagy in TME cells, we summarized the associations between the scores of these subclusters with prognosis and immune response based on publicly available bulk RNA-seq data. Obviously, EC patients with different domination of aggrephagy-related genes of the TME cells had huge prognosis differences in OS and ICB therapy response, especially for the CAFs, macrophages, and CD8+T cells, which disclosed that the crucial role of TME aggrephagy in further research.

This study has several limitations. First, our single-cell RNA-seq analysis of 36,227 cells from five EC tumor samples revealed aggrephagy-related heterogeneity in key TME cell types, the modest cohort size may weaken the generalizability of our findings. Second, our findings are primarily based on transcriptomic and histopathological correlations; functional validation of aggrephagy regulators at the protein level and their mechanistic roles in EC progression remain to be explored. Third, while TUBA1A was validated as a marker of the identified macrophage subcluster, this study did not directly establish a causal or functional link between TUBA1A+macrophages and aggrephagy. Future work should employ aggrephagy-specific assays to explore this relationship.

## Conclusion

In summary, we constructed a landscape of aggrephagy in EC, characterizing aggrephagy-related subtypes in CAFs, macrophages, T cells, and endothelial cells. We identified TUBA1A+Mac-C2 as a potential suppressor of lymph node metastasis in EC, providing a potential target for prognosis and therapeutic intervention in EC.

## Data Availability

The original contributions presented in the study are included in the article/**Supplementary Material**. Further inquiries can be directed to the corresponding authors.

## Supplementary material

Supplementary Figure 1 Overview of myeloid cell clustering and marker gene expression. **(A)** Dimensionality reduction clustering of myeloid cells. **(B)** Annotated marker genes for major myeloid cell types. **(C)** UMAP plots showing marker gene expression in detected myeloid cell types. **(D)** UMAP plot illustrating the four main cell type in myeloid cells.

Supplementary Figure 2 Overview of T/B cell clustering and marker gene expression. **(A)** Dimensionality reduction clustering of T/B cells. **(B)** Annotated marker genes for major T/B types. **(C)** UMAP plots showing marker gene expression in detected T/B types. **(D)** UMAP plot illustrating the seven main cell type in T/B cells.

Supplementary Figure 3 Overview of endothelial cell clustering and marker gene expression. **(A)** Dimensionality reduction clustering of endothelial cells. **(B)** Annotated marker genes for major endothelial types. **(C)** UMAP plots showing marker gene expression in detected endothelial types. **(D)** UMAP plot illustrating the three main cell type in endothelial cells.

Supplementary Figure 4 Survival analysis of TUBA1A in two databases. **(A)** Overall survival analysis of TUBA1A in TCGA database. **(B)** Disease free survival analysis of TUBA1A in Kaplan-Meier Plotter online platform.
