# Supplementary figures and images for: Single-cell sequencing reveals the role of aggrephagy-related patterns in tumor microenvironment, prognosis and immunotherapy in endometrial cancer
Source: Front Oncol. 2025 Mar 25;15:1560625. doi: 10.3389/fonc.2025.1560625 (PMC11975906; doi:10.3389/fonc.2025.1560625)

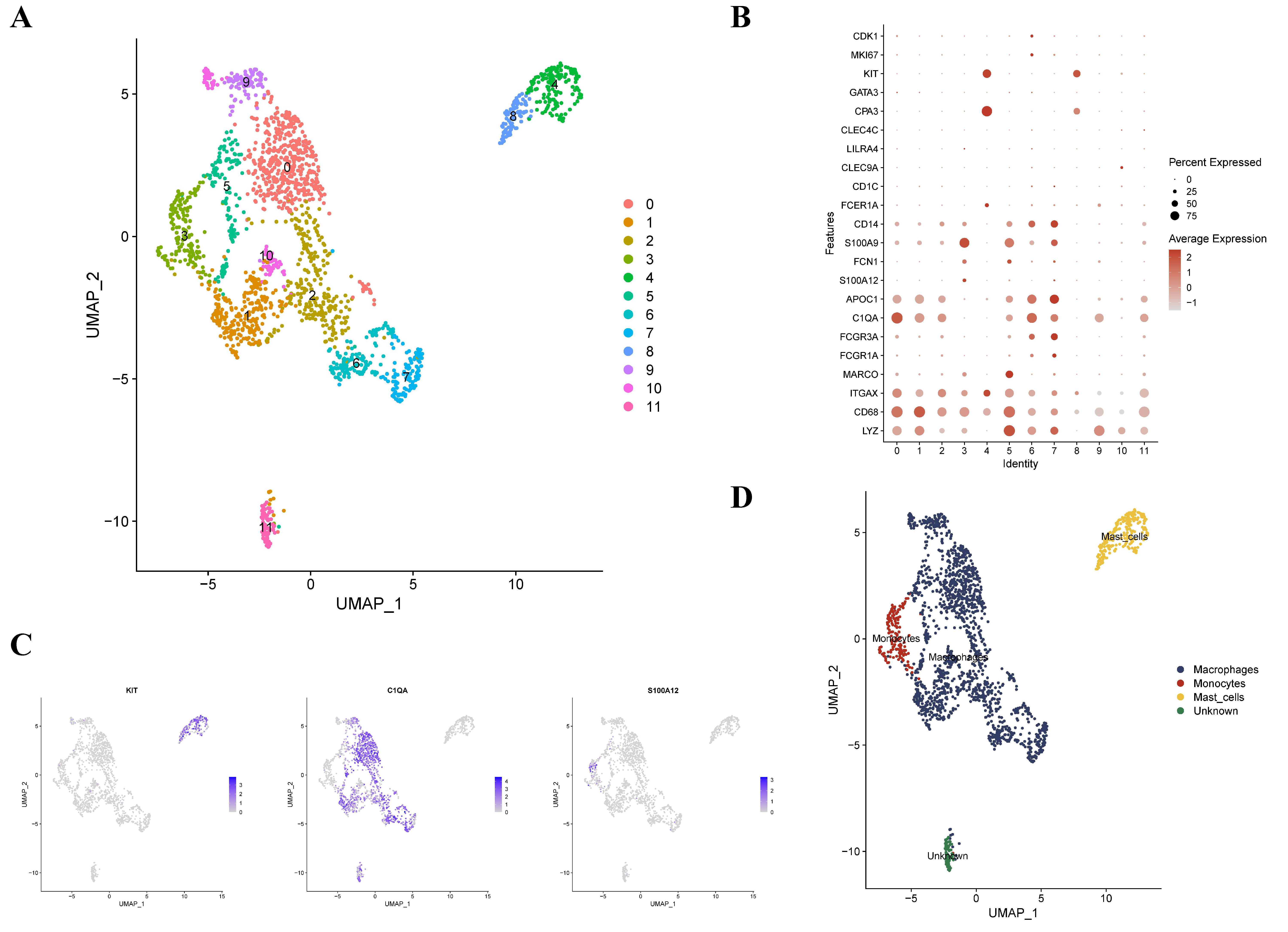

Supplement: Supplementary Figure 1 — Overview of myeloid cell clustering and marker gene expression. (A) Dimensionality reduction clustering of myeloid cells. (B) Annotated marker genes for major myeloid cell types. (C) UMAP plots showing marker gene expression in detected myeloid cell types. (D) UMAP plot illustrating the four main cell type in myeloid cells. [file Image1.jpeg]

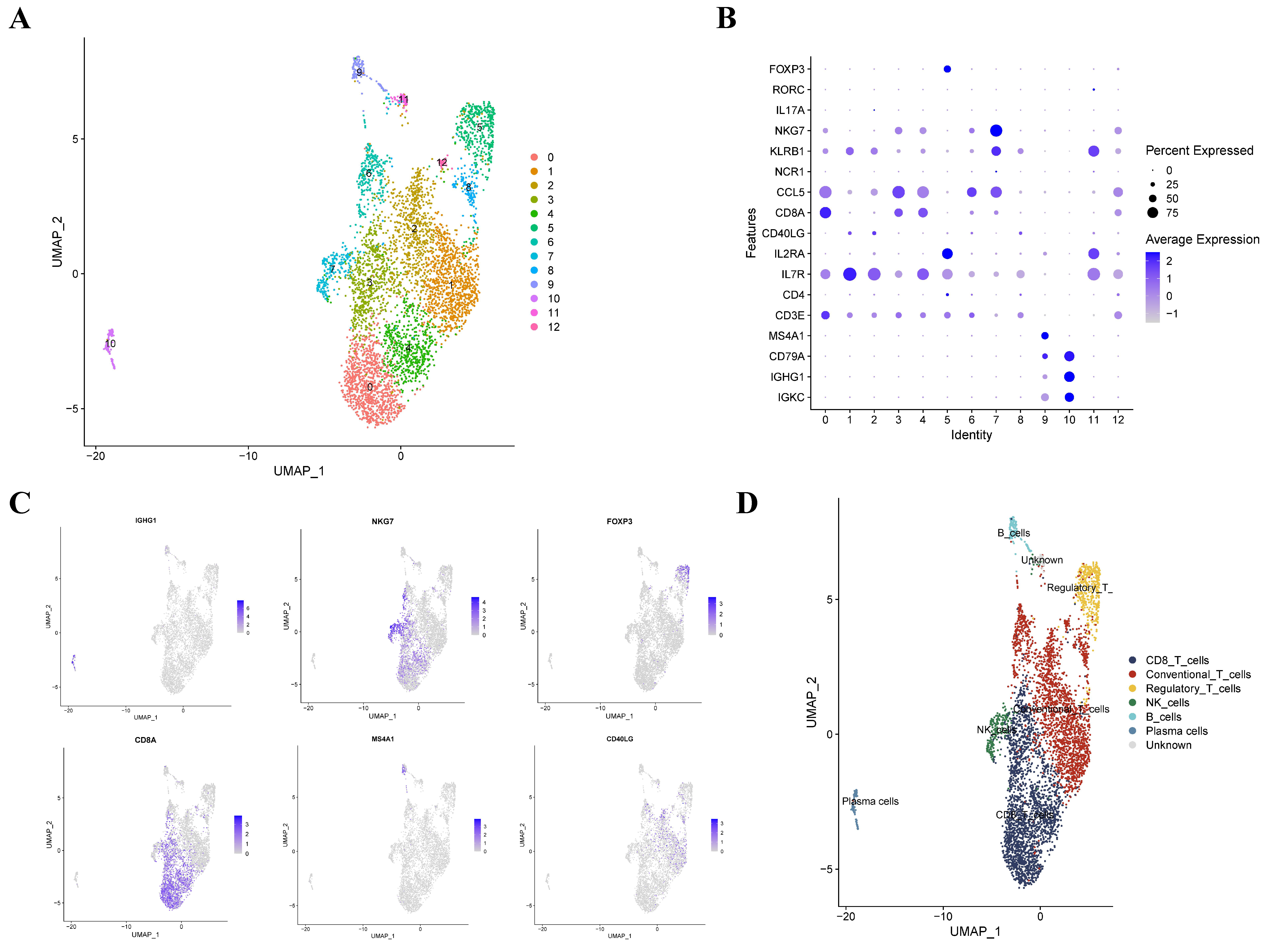

Supplement: Supplementary Figure 2 — Overview of T/B cell clustering and marker gene expression. (A) Dimensionality reduction clustering of T/B cells. (B) Annotated marker genes for major T/B types. (C) UMAP plots showing marker gene expression in detected T/B types. (D) UMAP plot illustrating the seven main cell type in T/B cells. [file Image2.jpeg]

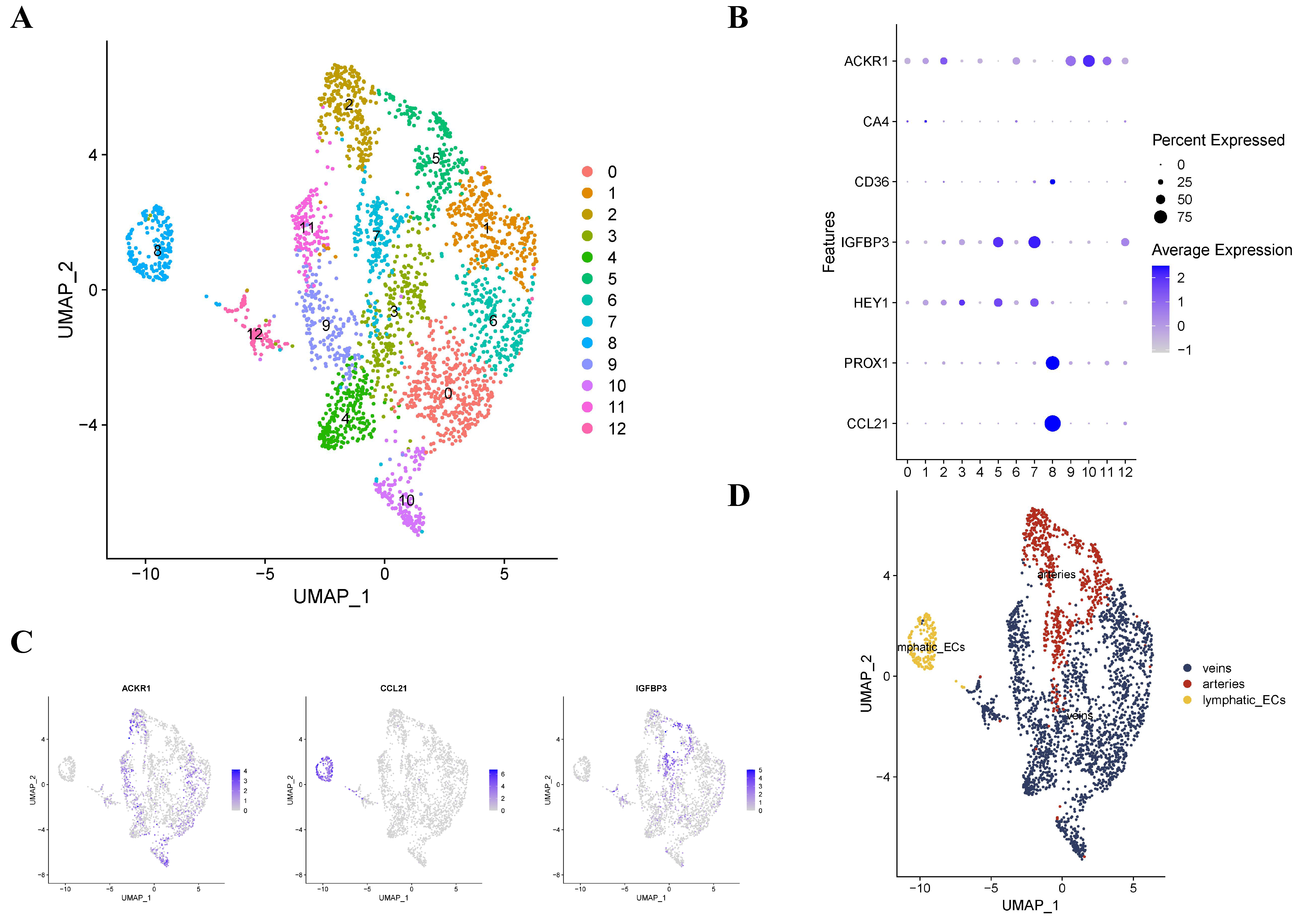

Supplement: Supplementary Figure 3 — Overview of endothelial cell clustering and marker gene expression. (A) Dimensionality reduction clustering of endothelial cells. (B) Annotated marker genes for major endothelial types. (C) UMAP plots showing marker gene expression in detected endothelial types. (D) UMAP plot illustrating the three main cell type in endothelial cells. [file Image3.jpeg]

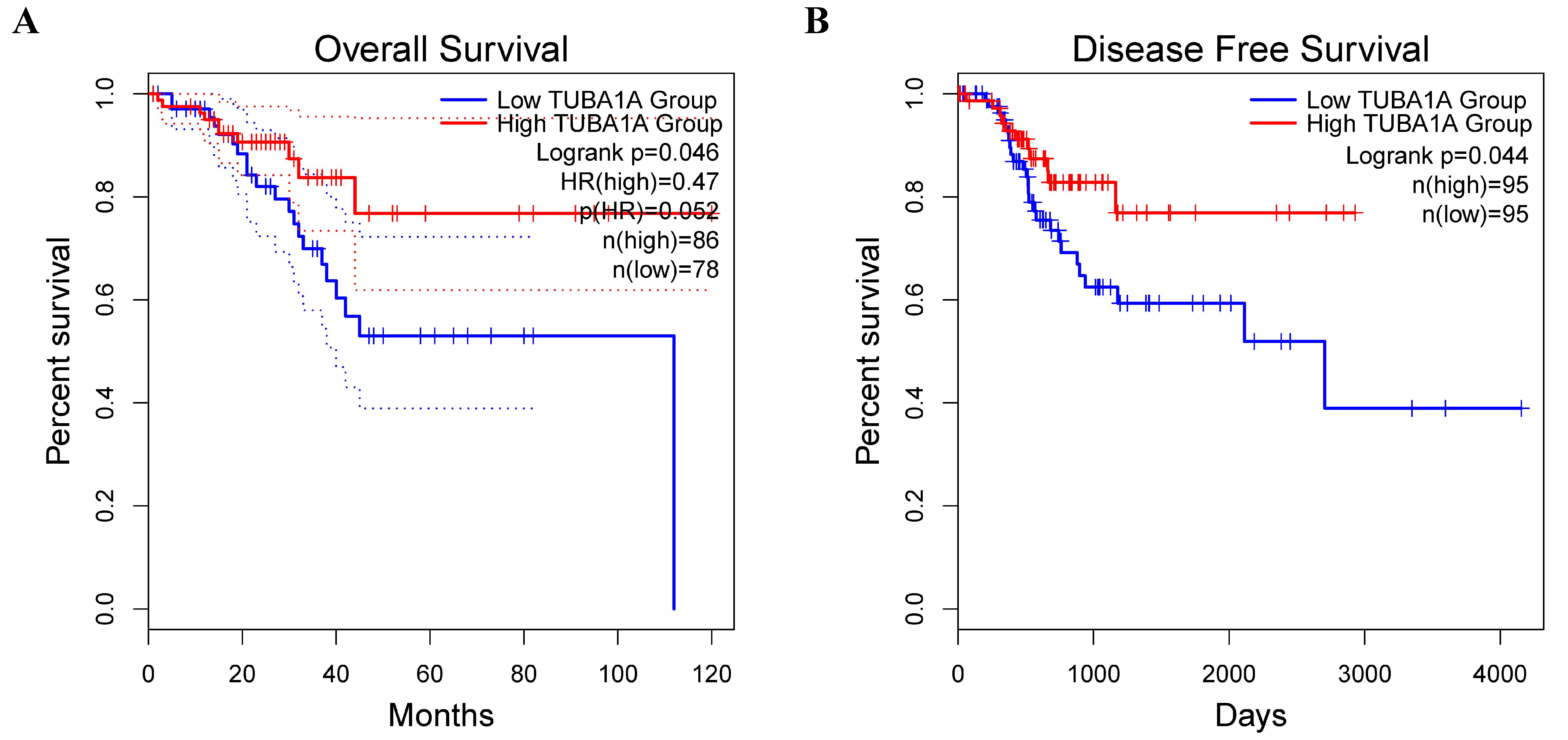

Supplement: Supplementary Figure 4 — Survival analysis of TUBA1A in two databases. (A) Overall survival analysis of TUBA1A in TCGA database. (B) Disease free survival analysis of TUBA1A in Kaplan-Meier Plotter online platform. [file Image4.jpeg]
