# Supplementary material for: Single-cell sequencing reveals the role of aggrephagy-related patterns in tumor microenvironment, prognosis and immunotherapy in endometrial cancer
Source: Front Oncol. 2025 Mar 25;15:1560625. doi: 10.3389/fonc.2025.1560625 (PMC11975906; doi:10.3389/fonc.2025.1560625)
Supplement: Supplementary file 5 [file Table1.docx]

supplementary file 1. The detailed information of included data

| **Dataset ID** | **Data Type** | **Samples (n)** | **Source URL** | **Reference** |
| --- | --- | --- | --- | --- |
| GSE173682 | scRNA-seq | 5 | [GEO Accession viewer](https://www.ncbi.nlm.nih.gov/geo/query/acc.cgi?acc=GSE173682) | Regner MJ, Wisniewska K, Garcia-Recio S, Thennavan A et al. A multi-omic single-cell landscape of human gynecologic malignancies. Mol Cell 2021 Dec 2;81(23):4924-4941.e10. PMID: 34739872 |
| TCGA-UCEC | Bulk RNA-seq | 583 | [GDC Data Portal Homepage](https://portal.gdc.cancer.gov/) | NA |
| GSE63678 | Bulk RNA-seq | 7 | [GEO Accession viewer](https://www.ncbi.nlm.nih.gov/geo/query/acc.cgi) | Pappa KI, Polyzos A, Jacob-Hirsch J, Amariglio N et al. Profiling of Discrete Gynecological Cancers Reveals Novel Transcriptional Modules and Common Features Shared by Other Cancer Types and Embryonic Stem Cells. PLoS One 2015;10(11):e0142229. PMID: 26559525 |
| GSE17025 | Bulk RNA-seq | 91 | [GEO Accession viewer](https://www.ncbi.nlm.nih.gov/geo/query/acc.cgi) | Day RS, McDade KK, Chandran UR, Lisovich A et al. Identifier mapping performance for integrating transcriptomics and proteomics experimental results. BMC Bioinformatics 2011 May 27;12:213. PMID: 21619611 |
| GSE115810 | Bulk RNA-seq | 24 | [GEO Accession viewer](https://www.ncbi.nlm.nih.gov/geo/query/acc.cgi) | Hermyt E, Zmarzły N, Grabarek B, Kruszniewska-Rajs C et al. Interplay between miRNAs and Genes Associated with Cell Proliferation in Endometrial Cancer. Int J Mol Sci 2019 Nov 29;20(23). PMID: 31795319 |
