# Supplementary material for: Single-cell sequencing reveals the role of aggrephagy-related patterns in tumor microenvironment, prognosis and immunotherapy in endometrial cancer
Source: Front Oncol. 2025 Mar 25;15:1560625. doi: 10.3389/fonc.2025.1560625 (PMC11975906; doi:10.3389/fonc.2025.1560625)
Supplement: Supplementary file 6 [file Table2.docx]

ARL13B

CETN1

CFTR

DYNC1H1

DYNC1I1

DYNC1I2

DYNC1LI1

DYNC1LI2

DYNLL1

DYNLL2

HDAC6

HSF1

HSP90AA1

IFT88

PARK7

PCNT

PRKN

RPS27A

TUBA1A

TUBA1B

TUBA1C

TUBA3C

TUBA3D

TUBA3E

TUBA4A

TUBA4B

TUBA8

TUBAL3

TUBB1

TUBB2A

TUBB2B

TUBB3

TUBB4A

TUBB4B

TUBB6

TUBB8

TUBB8B

UBA52

UBB

UBC

UBE2N

UBE2V1

VCP

VIM
